# Priorities of health policy: cost shifting or population health

**DOI:** 10.1186/1743-8462-2-1

**Published:** 2005-01-11

**Authors:** Jeff RJ Richardson

**Affiliations:** 1 Centre for Health Economics, Monash University, Melbourne, Australia

## Abstract

**Background:**

This paper is an edited version of an invited paper submitted to the Australian Health Care Summit on 17–19 August 2003. It comments upon the policies which have dominated recent debate and contrasts their importance with the importance of five issues which have received relatively little attention.

**Methods:**

Policy is usually a response to identified problems and the paper examines the nature and size of the problems which heave led to recent policy initiatives. These are contrasted with the magnitude and potential cost effectiveness policies to address the problems in five areas of comparative neglect.

**Results:**

It is argued that recent and proposed changes to the financing and delivery of health services in Australia have focused upon issues of relatively minor significance while failing to address adequately major inequities and system deficiencies.

**Conclusion:**

There is a need for an independent review of the health system with the terms of reference focusing attention upon large system-wide failures.

## 1 Introduction

The theme of this paper is that recent and proposed changes to the financing and delivery of health services in Australia have focused upon issues of relatively minor significance while failing to address adequately major inequities and system deficiencies. An intriguing question – not discussed in the paper – is how such drastic failures could continue year after year with little comment and no decisive policy commensurate with the magnitude of the problems. The political-sociological answer to this question is undoubtedly complex and contentious. The failure may (or may not) be attributed to understandable, even unavoidable obstacles arising from Australia's social history. Nevertheless it is important to be aware that these failures exist.

The current state of our health services could justifiably be described as a 'silent crisis'; service delivery is highly inequitable and inefficient; patients are dying unnecessarily and avoidable medical errors are imposing huge financial and human costs on the community. While this occurs, health policy at the political level has focussed upon cost shifting between the States and Commonwealth, between public and private sectors and between the well off and the poorer members of society. Reforms addressing the larger issue have progressed at glacial speed, relative to what is achievable, or they have stalled altogether.

In Section 2 there is a brief overview of the economists' analytical framework in order to introduce two preliminary issues, viz, the role of social values in health system reform and the constraints created by the limited availability of resources. Two commonly made but wrong inferences from this latter constraint are discussed. Section 3 is concerned with recent policy and, in particular, the changes to private health insurance (PHI) which have been introduced since July 1997. In contrast with the *relatively *inconsequential (and possibly negative) impact of these policies, five major problems are outlined in Section 4, each of which has received insufficient or no attention.

Policy implications and options for future policy are discussed and highlighted throughout the paper. In the final section I argue that the optimal health system – entirely public, largely private or one of the myriad combinations between these polar options – is the system which is most likely to address systemic failures. The most important changes to achieve the optimal system may have less to do with the public-private mix of services, or even funding, than with the extent to which these failures are addressed within any of the conceivable health systems of the future. This, in turn, may depend upon the willingness to create appropriate economic and other incentives.

## 2 Resources, values and the economic framework

The discipline of economics provides a framework for the analysis of options which is based upon a comparison of costs and benefits. If social welfare is to be maximised then the logic of the framework must be adopted explicitly or implicitly. The framework focuses attention upon the benefits which might be obtained when resources are used in a particular way, and the benefits which might have been obtained if they had been used somewhere else – the (opportunity) cost of using those resources in the chosen way. Social wellbeing is maximised when the benefits exceed the (opportunity) costs in every setting and on every margin where choice is possible.

While this statement is tautologically true, the focus upon choice highlights two important facts. First, choices generally do exist; the economy is flexible and choices are driven by individual and social preferences. Technical inevitabilities are rarely encountered. Second, and more fundamentally, it is necessary to define 'benefits'. In principle, the abstract framework is consistent with an almost unlimited number of value systems. For example, in the context of an intensive care department with limited capacity, benefits and costs might be measured by lives saved and lives lost. In this simple example the cost benefit formula would translate into a policy of providing ICU beds to those most likely to live.

Social objectives in the health sector are clearly more complex than in most other settings and the nexus between objectives, policies and the optimal health system is more problematical. Nevertheless, an important conclusion from this framework (which needs constant repetition) is that there is not a single 'best' health system; rather, there are various options which are more or less consistent with different social goals.

This conclusion is illustrated in Table 1 using two highly simplified but archetypal social objectives, viz, the egalitarian desire for equal access to health or health services and the social objective of maximising individual choice. As shown, the first of these objectives is more easily achieved through a compulsory public system with defined benefits and constrained choice. The second objective is most likely to be achieved in a less constrained and more competitive private system which responds to individual preferences, as described and generally prescribed by economic theory for less complex markets and social objectives.

**Table 1:** The relationship between choice, values and the optimal health system

**Objectives/Social Values**	**Option which maximises likelihood of success**
Equalise access, outcome	Universal (monopoly) Public Insurance/Financing
Maximise choice; diversity + safety net Optimise the max of these 2 objectives	Pure private (competitive) scheme
Both of the above	Mixed public-private scheme

***Conclusion 1:**The form of the optimal health scheme depends upon social objectives and disagreement about these translates into differences with respect to the funding and delivery of health services.*

While the two objectives in Table 1 are archetypes, they broadly correspond with two important but conflicting 'world views'; that is, with different ethical beliefs about the appropriate supply and financing of health services. The social values underpinning the competitive market model are well articulated and well labelled. Its 'liberal' or 'libertarian' value system emphasises the importance of individual responsibility and freedom of choice. It is the prevailing value system in many aspects of life in a democratic society and there is commonly a presumption that, in the absence of some compelling argument, liberty and choice should be maximised. In economic theory this objective is equivalent to the goal of maximising 'utility' and the Welfarist theory of 'Social Welfare'. Even those expounding liberal values, however, generally believe that some constraint upon choice, in the form of compulsory taxation, is justifiable to finance a limited number of public goods and that at least basic medical services should be provided for the medically and financially indigent. With this 'world view' fairness generally equates with a vertical redistribution of income to help the most needy.

In contrast, the value system underpinning the public model is less clearly articulated (at least in Anglo Saxon countries). The financing and provision of services to the entire population is often characterised as 'middle (and upper) class welfare' and contrasted with the less intrusive 'safety net welfare' which is all that is required to help those who cannot help themselves. This interpretation of egalitarianism does not, however, correctly represent the values which underpin the public health insurance system. These are nicely described in a report of a commission of enquiry into Canadian Medicare as follows:

'Canadian Medicare is far more than just an administrative mechanism for paying medical bills. It is widely regarded as an important symbol of community, a concrete representation of mutual support and concern... it expresses the fundamental equality of Canadian citizens in the face of death and disease... as the Premier of Ottawa pointed out... "There is no social program that we have that more defines Canadianism"' [1].

The social value or world view embodied in this quotation does not correspond with the simple notion of assistance for the indigent. Rather it corresponds with a desire to 'remove health and health care from the economic reward system' in the same way as all citizens are, in principle, given equal protection by the law. A close analogy is the desire to have public parks which may be accessed by all members of the community without payment. The objective is not a redistribution of income or the provision of a safety net. Rather, with this view, access to public parks is one of the consequences of belonging to the community; it is a shared benefit and, as such, engenders a feeling of sharing, participation and belonging. The concept is closely linked to the notion of 'social capital' which 'accumulates' with an increase in communal sharing and participation. Pay parks are possible, but a fully informed community might reject this option. Its citizens may wish to live in a community where the Arts flourish, where its sportsmen and women are a source of national pride, where parks are free for all citizens, where an acceptable standard of living is guaranteed after retirement and where all citizens have access to the same range of medical services. In European countries the term 'solidarity' is used to describe this value system. Unfortunately, in English, there is no commonly used and understood word for the concept. ('Communitarianism' is the closest translation.) The consequence of this is a degree of confusion in the expression of social values as both sides of the debate attempt to appropriate the word 'equity' to support their world view. It is clearly desirable that the debate should not be derailed by linguistic ambiguities.

***Conclusion 2:**Health policy should be informed by a careful evaluation of the social values held by different groups of the community with respect to different elements of the health system.*

While it is ultimately the responsibility of the government to decide which of these values should be embodied in policy, it is desirable for the government's decision to be informed by evidence concerning the community's values and the strength of preferences for different values systems by different groups in the community. In his influential book 'The Power of Public Ideas' Robert Reich emphasises the importance of 'discovery', or the process of getting the public to articulate these values [2]. To date, neither this nor research into health related social values has been carried out satisfactorily in Australia.

***Conclusion 3:**The choice between public and private funding of health services depends upon social values and, in particular, the strength of liberal-libertarian versus solidarity-communitarian values as they apply to the health sector.*

Returning to the first defining characteristic of the economic framework, the emphasis upon choice conflicts with two commonly held technocratic beliefs about the inevitability of particular 'problems'. First, the common perception that the country cannot, or shortly will not be able to afford health services is unambiguously false, at least in the foreseeable future. In the USA, per capita expenditures are about double the Australian level and the US Health Care Financing Agency (HCFA) has projected a doubling of these expenditures relative to GDP by 2030. This is technically possible. The relevant question is whether or not we obtain commensurate benefits from these expenditures and if, as a society, we chose these benefits in preference to the benefits foregone. Taking an extreme example, if Australians could spend 25 percent of GDP upon health services this option would probably be embraced enthusiastically if it resulted in an illness free life expectancy of 120. Optimal expenditures are entirely a function of the benefits we obtain and are not driven by technological imperatives.

***Conclusion 4:**There is no immediate limit to the optimal level of health expenditures. It is technically possible to increase present expenditures very significantly. The optimal level depends upon the costs and benefits of the various health services. Increased health expenditures should be enthusiastically embraced if they improve health and health related objectives sufficiently. Technocratic arguments asserting the economic impossibility of increased health spending or increased public funding are unambiguously wrong. At best they are based upon unstated political/ideological assumptions and not economic arguments.*

A similar argument applies to the second non-problem – the impossibility of funding health services through public taxation. Arguments of the form 'the Government can't afford to pay' are also unambiguously false. The country which can afford to finance health expenditure from private health insurance can also afford to pay an equivalent amount through taxation and some have argued that PHI is, itself, a form of privatised tax. The government share of the health bill is smaller in Australia than in most developed countries. Likewise, taxation is relatively low. This implies that Australia could significantly raise its level of public funding without exceeding the tax burden which is presently experienced in most comparable countries. More fundamentally, however, the form of financing for health services is flexible and is again a matter of social choice. It is likely that this choice will be influenced by the relative costs and benefits arising from the choice, but in the health sector the known costs and benefits associated with public and private health care are not compelling. Privately funded health care is often a little more expensive, but countries with a strong preference for liberal-libertarian values might sensibly opt for a relatively larger private scheme even if it is more expensive. The principle of paying more for what is wanted should not be controversial!

***Conclusion 5:**The balance between public and private sources of revenue for a health service should be determined premanually by the social philosophy of the country. There are no compelling technical or economic constraints on the freedom of sound choice.*

## 3 Recent policy issues

Public debate has recently focused upon three 'problems', namely the high and rising cost of pharmaceuticals, the declining rate of bulk billing by General Practitioners (and particularly amongst health care card holders) and the declining number of people purchasing private health insurance. The comments below do not purport to be an exhaustive analysis of these subjects but are included to contrast the subject matter of the policy debate with the more substantive problems which will be discussed in the following section. Pharmaceuticals and PHI are discussed more fully in Richardson and Segal [3].

### Pharmaceuticals

Pharmaceuticals are included in the Pharmaceutical Benefits Scheme (PBS) after a detailed review of their effectiveness and cost effectiveness (see Salkeld et al 1998 for a description [4]). This process does not, by itself, reduce expenditures. Rather, it ensures that drugs whose effectiveness is low in relation to their cost will not be adopted. Expenditures will be lower if the manufacturers of relatively cost ineffective drugs reduce their prices to increase the likelihood of their inclusion in the PBS. However, this may be offset (more or less) if drug companies increase the price of new highly cost effective drugs as they know that the PBAC is aware of their cost effectiveness. Cost effective drugs may also be overused if doctors prescribe them for purposes or at thresholds not tested before their introduction. Partly for this reason the PBAC has sometimes negotiated a price-volume trade-off – if drug use exceeds the initial expectation then the agreed price is lowered.

These measures have not contained pharmaceutical costs. In view of rising drug prices in other countries it is likely that this is primarily or entirely attributable to the high cost of new generation drugs and is not attributable to policy. Nevertheless, expenditures have risen and in recent years copayments have been progressively increased in order to reduce government expenditures through the PBS. However it is not clear that the use of copayments, and particularly in a single sector, will have an overall beneficial effect. First, as demonstrated by the Rand Experiment copayments reduce demand somewhat (elasticities are low but not insignificant) [5]. There is a disproportionate effect upon low income households [6] which, in this case, implies low income non health care card holders. There is little evidence that lower income patients will discriminate between effective and less effective drugs and at least one study suggests that, perversely, the greatest impact will be upon life saving drugs which have a relatively small immediate impact upon symptoms [4,7].

Secondly, relatively larger copayments in one sub-sector violates a fundamental principle for achieving allocative efficiency; viz, a 'level (financial) playing field' between alternative products/interventions. Violation of this condition increases the likelihood that less cost effective services may be used because of the distorted price signals. Allocative efficiency depends upon relative prices rather than the existence of copayments per se. More specifically, high pharmaceutical prices at the point of service will encourage the use of potentially more expensive interventions including hospital services if a copayment results in the deterioration of a patient's health.

Thirdly, the effects of copayments on national health expenditures will be relatively small. Wealthy individuals will simply pay the copayment and health care card holders will be largely shielded from them. The most important effect of the copayment is that it will shift costs from the taxpayer to the patient; that is, its major effect will be upon the distribution of income with the healthy-wealthy taxpayer gaining at the expense of the less healthy-less wealthy.

In 1960, pharmaceutical costs represented an estimated 22.3 percent of health expenditure. By 2002 they were 13.5 percent of the total. The comparison indicates that the composition of expenditures may vary significantly through time and, taken out of context, focus upon a single sub-sector may be misleading. Cost control requires a full system perspective and there is no optimal level of pharmaceutical expenditures which is independent of the cost of alternative services. The problem is, in part, attributable to the program structure of delivery and financing which treats pharmaceuticals as an independent program as distinct from an input into an overall treatment regime.

***Conclusion 6:**Reliance on copayments for the control of pharmaceutical expenditures is probably an inappropriate policy as it violates an important principle for achieving allocative efficiency. A more global approach to health policy analysis and reform is needed.*

### Bulk billing

Since the introduction of Medicare the percentage of GP attendances bulk billed rose from 52.5 percent in 1984/85 to a maximum of 80.6 percent in 1996/97, then fell to a low of 66.5 percent in December 2004. The measures discussed below appear to have arrested this trend and by March 2004 bulk billing had risen to 68.7 percent of GP attendances [8].

The chief problem the reforms were designed to overcome was the possibility that the falling level of bulk billing may have jeopardised access to health services by pensioner/health care card holders and other low income households. Importantly, data did not exist to demonstrate that this was, indeed, a problem and that bulk billing or service use by this group had declined by the average or near average percentage. That is, the existence and quantitative significance of the 'problem' were not documented.

The recent 'Medicare Plus' package introduced a potentially important structural change. Rebates for bulk billed/health care card holders were separated from the rebate to the general public thereby allowing the separate treatment of the two groups. To encourage the bulk billing of the first group the benefit was increased and bulk billing doctors were permitted, for the first time, to direct-bill the Health Insurance Commission for general patients while simultaneously charging an 'over the counter' extra payment, a measure widely perceived as encouraging an increase in fees as patients will commonly equate the relatively small copayment with the total fee.

These measures have two important effects. First, the probable increase in general fees allows the benefit payable for GP bulk billed pensioners/health care card holders to be at a lower level than would occur than without the effective cross subsidy from increased general fees. Secondly, the government can preserve 'equity' (ie bulk billing the needy) by adjusting only the pensioner/health care card holder benefit, while simultaneously allowing a deterioration of the general rebate. Cost shifting would have been further facilitated by an earlier agreement to allow private health insurers to reimburse medical expenses above the schedule fee as part of an agreement with doctors to remove out-of-pocket costs. But this proposal was rejected by the Senate.

In sum, there is now a structure which facilitates cost shifting from the public to the private sector. There are two important caveats to this conclusion. First, a rebate structure which facilities additional cost shifting does not imply that government will use this option in the short or long run. Secondly, the effect of additional cost shifting will be mitigated by an additional element of the Medicare Plus package, namely, the introduction of a 'safety net' which reimburses 80 percent of out-of-pocket, out-of-hospital costs including billing above the schedule fee once family expenses reach $1,000 (or $500 for a health care card holder's family). The long run effect of this latter cost off-set is difficult to assess. As reported in the announcement of Medicare Plus the expected cost of the safety net in its first three years would average $89 million p.a. as compared with likely out of pocket medical expenses of about $1,400 million. in 2004/05 (extrapolated AIHW) [9]. Even allowing for a probable cost overrun the amount is not large. Nevertheless, the 'safety net' offers additional protection to those who would be most affected by additional cost shifting; that is, this policy also helps create a structure where equity, defined in terms of need, may be reconciled with increased cost shifting.

However, the most significant feature of these changes to the reimbursement formula for medical expenses is that they deal with *relatively *'small order' issues.

Under Medicare, pensioners/health care card holders with significant health problems have access to emergency and outpatient facilities at public hospitals. Poor access to these will increase inconvenience and queuing and, in an unknown number of cases, result in poorer health. But this more serious outcome is likely to be *relatively *infrequent as the majority of this group have always been bulk billed while others will have satisfactory access to hospital based care.

***Conclusion 7:**Proposed and actual policies towards pharmaceutical expenditures and bulk billing have had a common element. Both represent immediate or potential cost shifting from the government to the public and any net reduction in societal expenditure because of a reduction in patient initiated services will have potentially adverse effects upon the health of the 'near poor'. However, the financial effects of the policies are relatively small and the indirect adverse effect upon health is likely to be correspondingly small.*

### Private health insurance (PHI)

The role and regulation of PHI after the introduction of compulsory national health insurance has been ambiguous and anomalous. Since Medicare was introduced as the vehicle for achieving fairness in the financing and delivery of health care it has never been clearly stated why PHI should be closely regulated to achieve fairness in he sub-group of the population which elects to purchase PHI and why it is regulated in a way which inhibits effective competition. But these are the effects of Australia's community rating and reinsurance requirements respectively.

In the last one and a half decades there has been an ongoing concern that declining levels of private health insurance have had an adverse effect upon the public hospital system and that 'PHI reform' has been needed to solve this problem. The argument is summarised in the following (constructed) quotation:

'Private health insurance was caught up in a downward spiral caused by the adverse selection identified in the Productivity Commission report [10]. 'Rising premiums triggered by increasing costs induced low risk members to withdraw. Without their cross subsidy of high cost members, premiums were forced to rise again, which further increased premiums, which induced further departures, etc.

"*PHI is primarily purchased to cover the costs of private hospitalisation. Consequently, as PHI declined through time, fewer patients have been able to afford the cost of private hospitals, and this has created an excess demand for public hospital beds. The increasing length of queues is a confirmation of this problem*".

While plausible, the account of history in the second half of this argument is wrong. Between 1985/86 and 1999/00 private hospitals increased their share of admissions by 32.4 percent from 25.9 to 34.3 percent of the total. The share of bed days rose from 21.9 to 28.1 percent of the total or by 28.3 percent [3]. These simple and readily available statistics unambiguously contradict the conventional wisdom propagated by the media and some politicians. It is true that queues in public hospitals have increased, but this is attributable to the increasingly draconian budget caps upon public hospitals throughout the 1990s. That is, queuing in the public sector has been primarily a result of supply side and not demand side factors. (Excess demand for beds cannot explain the closure of wards that occurred in many public hospitals!) The simplicity of the statistics contradicting the conventional wisdom calls into question the analytical capacity of many media commentators (and some independent commentators)!

***Conclusion 8:**Media analysis of the relationship between PHI and queuing in the public system highlights an important system failure; viz, the failure of the media to exercise rudimentary critical skills in their analysis of PHI and hospital queuing.*

Even if PHI membership had fallen sufficiently to have reduced the share of private hospital admissions by almost a third, the balance of public and private hospital separations in 1999/00 would have been similar to the share in 1985/86. This suggests that it was the events summarised in the first half of the quotation which were of concern at the government and departmental level and that PHI membership and the public-private mix of health financing and delivery were explicitly or implicitly the real target of health policy.

In response to the 'problem' of falling PHI and the demand side problem for public hospitals (which did not exist!), the government introduced three enduring policy changes. In July 1997 individuals with an income above $50,000 and families with a combined income above $100,000 who did not purchase PHI became liable for a 1 percent tax surcharge upon their incomes. In December 1998 a 30 percent rebate on PHI premiums was introduced. (In August 2004 a selective increase in the rebate was foreshadowed); and in September 1999 lifetime community rating was enacted which has the effect of reducing future premiums for those who have held PHI from the age of 30. Discounts apply at any age between 30 and 64; 30 is the age of maximum discount. Failure to enrol by this age will result in a higher premium if PHI is subsequently purchased.

The evidence demonstrates that these policies, and particularly the last policy, have succeeded in increasing PHI membership. However it has created an industry which, along with the platypus and echidna could be Australia's entrants into the world's strange but true contest. Australia would almost certainly win – even without our egg laying marsupials. First, the surcharge results in a negative price for the wealthy. Individuals and families above the income threshold avoid an increasingly large tax payment as their income rises; that is, if they purchase PHI they will have a greater income at the end of the year than the individuals and families above the threshold who do not buy PHI. This is analogous to supporting the automobile industry by placing a surcharge on wealthy families who fail to buy an Australian car. It would be difficult to find any other support scheme which uses the income tax system to coerce the purchase of a particular product. It would be equally difficult to find a produce where the price is negative.

Secondly, (and predating the recent legislation) the use of PHI to cover hospital bills generally results in a greater, not smaller, out of pocket expense. Public hospitalisation is free. Those with PHI commonly pay a copayment. (There is a perverse equity in these two anomalous outcomes. The wealthy are paid to take PHI but financially penalised if they use it!)

Third, lifetime community rating also involves a leap of faith. The 30 year old must believe that public policy will remain stable for 50 years – a belief that should have been challenged when both parties announced changes in the 2004 election campaign. However lifetime community rating also has a bizarre dimension. Insurance is generally purchased to reduce risk and uncertainty. In the health sector these arise because of the risk and uncertainty of ill health and the cost of medical care. Prior to lifetime community rating this uncertainty depended upon possible events in the following 1–3 years. After the change it depended upon the next 1–30 years. Events in 30 years are more uncertain than events in 3 years and, consequently, the legislation increased risk and uncertainty. The perceived risk was amplified by the publicly financed marketing of private insurance. Predictably, people responded to the increased uncertainty by buying more insurance. Thus, to encourage the uptake of insurance the government increased the very thing insurance is designed to reduce, viz, risk and uncertainty.

A final anomaly which predates the recent PHI reforms is that private health insurance in Australia leaves the consumer with residual risk. Particularly for ancillary services, most benefits are capped and out of pocket expenditures rise with the price of the service. This pattern of benefits is exactly the opposite of the structure of benefits which will maximise the patient's 'expected utility' [11].

Taken together, the changes introduced since July 1997 have created an extraordinarily complex and perverse set of incentives. The ethical basis of the free market and the liberal-libertarian model is that choices should be determined by individual's preferences in relation to real (opportunity) costs. The surcharge subverts this process and coerces choice and the strength of the coercion is based upon economic class. There is no justification for this in the economic theory of the efficient market.

Despite this conclusion, it is a legitimate function of government to determine the balance between public and private delivery financing and the distribution of health care costs. As described earlier the preference for private sector funding and provision (*albeit *in the context of compulsory core insurance) is consistent with a legitimate and defensible world view, viz, the liberal-libertarian belief that in a free society individuals should be encouraged to take responsibility for their own lives. In particular, the 30 percent subsidy is the orthodox approach to encouraging an industry which has a special claim for protection and, private health insurance does not compete on a 'level playing field': it competes with a public sector which is free at the point of service.

There are, however, two important caveats. First, and as noted above, the measures taken have destroyed any nexus between potential benefits and the price paid by wealthy individuals. Secondly, the 'product' is unlike usual insurance where the benefit – a payment after an adverse event – does not impinge upon other individuals. PHI is purchased to avoid queues and to select the best possible doctor. With fixed capacity, the avoidance of queues by one person imposes a longer queue on another person – there is a 'negative externality'. Selection of the best doctor reduces the access to these doctors by public patients. Consequently, the important debate should be about the 'right' to purchase preferential care *at the expense of those who do not have PHI*. In a liberal democracy there is a presumption that individuals may spend their own income as they wish. For the individual there is probably no more important context for exercising this 'right' than in the context of preserving life and its quality.

There is, therefore, a head-on-head conflict between the liberal-libertarian 'right' of the individual to spend his or her income on health care and the communitarian-solidarity based 'right' of each individual in a community to have equal access to high quality medical care. The latter goal must necessarily be achieved by imposing some constraint upon income-based preferential care to a particular group in the community. In Australia the constraint has been a de facto financial penalty for seeking priority care. Until the advent of engineering of negative prices for the wealthy, the purchase and use of PHI resulted in 'double payment' for some services, first via taxation and the Medicare Levy and secondly by premium paid for PHI. This 'penalty' still exists for lower income individuals and households.

***Conclusion 9:**The public debate over Private Health Insurance has been misleading. The contentious issues do not only concern the most effective way of ensuring access to health care (with the erroneous presumption that public monies not spent on health services represents wasted resources). Rather, the contentious issues include the 'right' or otherwise of the individual to spend their own income on whatever they wish without coercive financial penalties and the consequences for the remainder of the community when one group jumps the queue and has priority access to the most experienced doctors.*

### Distributional effects of recent policies

PHI policy has been consistent with the other policies discussed above in one important respect. The policies are likely to have a relatively small effect upon the *delivery *of health services. Rather, they are about financing care and the public-private balance in the health system. The balance, in turn, affects the distribution of health care costs between different households. Copayments shift costs from the government to patients. As government payments are met by progressive taxation, copayments redistribute the cost of health care from wealthy-healthy taxpayers to the less healthy, less wealthy. Additionally, as copayments have a disproportionate effect upon the use of services by low income households, the proportion of the government subsidy returned to high income households rises as copayments rise.

The redistributive effects of PHI are more complex. Low income households which purchase PHI unambiguously pay more for hospital and health services. Their taxes and the Medicare surcharge are unchanged and the purchase of PHI leaves them out of pocket. For higher income households, which are liable for the PHI surcharge, the effects are conceptually ambiguous as they depend upon the assumption made about the surcharge in a counterfactual world in which PHI did not result in a surcharge exemption. The surcharge was created specifically to permit an exemption for those who purchased PHI and the removal of the exemption might therefore be accompanied by the removal of the surcharge.

Finally, and as noted earlier, the proposed changes to bulk billing represent a structural change which will facilitate the transfer of medical insurance costs from the public to the private sector.

***Conclusion 10:**Recent and foreshadowed legislative policy initiatives with respect to bulk billing, copayments and PHI, all concern the financing of health services. Recent policy has been introduced with measures to mitigate the impact upon the most needy and, in the case of medical rebates, in a way which accommodates the popularity of bulk billing. Nevertheless, a common feature is that each of the proposed or implemented policies assists with the creation of a structure which facilitates the transfer of expenditure from the public to the private sector. If this occurs, it will reduce the cross subsidy from healthy wealthy to unhealthy less wealthy households. This effect occurs immediately with copayments. The transfers are more complex in the case of PHI.*

## 4 Five major problem areas

The five issues below have two common elements. First, they are directly concerned with health services and not the distribution of costs and incomes. Second, they have been almost ignored in the public policy debate despite being problem areas where there are quantitatively large inefficiencies and a corresponding potential to increase health outcomes and/or increase the overall cost effectiveness of the system.

### Efficacy, effectiveness and social objectives

In common with all other health systems many, and probably most, of the services provided in Australia have not been adequately evaluated and there is no ongoing process for the elimination of cost ineffective services. In 1987 Chassin et al [12] estimated that between one third and one half of coronary services in his study were 'inappropriate' in the sense that they had no beneficial, or a detrimental affect upon health. An additional one third to one half of the procedures considered had equivocal benefits. Likewise, Brook [13] estimated that 51 percent of angiography and 42 percent of coronary artery bypass graft procedures were unnecessary. Other studies by the US Health Care Financing Agency and the OECD have likewise concluded that only a small number of services have been evaluated for efficacy [14]. In Australia Segal [15] demonstrated that in the context of diabetes the cost of obtaining a life year varied from $70,000 (drug therapy) to $2,400 for behavioural programs. Comprehensive diabetes care was estimated to have a negative cost per life year; ie the program saved life and saved cost.

With respect to this issue, the Australian record is comparatively good. It has led the world with the introduction of mandatory economic evaluations for the drugs and services to be subsidised through the PBS and the Medical Benefits Scheme respectively. However the failure of other countries does not indicate that Australian procedures are satisfactory. The overwhelming majority of the services which were accepted before the introduction of mandatory economic evaluations have not been assessed and, with the passage of time, there is a need for the reassessment of services and drugs.

A related problem is that drugs or services which are efficacious when used appropriately in a random control trial may be used inefficiently with inappropriate patients who do not have the clinical indications of the patients in the trial. Gold standard medical care requires that cost effective therapy, drawing upon evidence-based medicine, should be the norm and not the exception in medical practice. Cost effectiveness should be determined by a broad ranging comparison of options form across the full range of potential interventions; that is, comparator interventions should take account of substitute services from across the entire health sector. This does not presently occur. Guidelines for the PBS require a comparison between drugs and exclude comparison with lifestyle/behaviour change programs.

***Conclusion 11:**The scale of present evaluation activities is inadequate. In an industry absorbing 9 percent of the GDP – the country's largest industry – there should be ongoing and large scale evaluation and re-evaluation of the cost effectiveness of the services provided. Evaluation should be based upon a comparison with the full spectrum of substitute services. A failure to do this almost certainly ensures that there will be widespread and significant allocative inefficiency in the level and mix of services.*

Implementation of evidence based practice will inevitably be resisted by service providers whose practice and income may be adversely affected. Consequently, provider education for a 'culture change' is likely to be a slow and ineffective vehicle for change. In contrast, once the desired practice pattern has been determined, financial incentives to encourage this form of practice may be implemented relatively quickly and do not involve direct coercion. The economics literature is replete with examples where such incentives have significantly altered provider behaviour [16-19]. Australian examples include the use of GP incentives to bulk bill; to undertake rural practice and to increase child immunisation. DRG based financial incentives were spectacularly successful in increasing hospital throughput in Victoria in the early 1990s [20].

***Conclusion 12:**Reimbursement formula for service provision should include financial incentives at all levels of service delivery for the use of the most cost effective therapies.*

The more general principle which should, but does not, permeate the market for health services is that the willingness to pay for services should vary with social objectives, a principle which is successfully incorporated in simple competitive markets when social and individual objectives are identical. As one example where this principle has recently and successfully been used, if society wishes to encourage bulk billing (which clearly benefits patients but lessens provider control over their incomes) then the benefit for services that are bulk billed should be increased relative to the rebate for other services and the differential increased until the target of bulk billing is achieved. Likewise, if society wishes providers to adopt evidence based medicine, hospitals to install clinical pathways, some procedures to be encouraged and others discouraged, then payment for the desired service or process should be increased relative to the reward when these services or activities do not occur.

At the level of the individual service, recent research has demonstrated health-related social objectives which are often broader than the minimisation of morbidity and mortality, for example, preferential treatment for particular age groups, particular classes of patients and health benefits [21,22]. Presently these additional 'social preferences' are ignored. This is unsurprising as the research to identify and measure them is still underway. However, where these preferences are significant and consistent, payments should eventually be adjusted to ensure that they are achieved.

***Conclusion 13:**The payment of service providers should incorporate the principle that society should pay for what it wishes to have in accordance with its social objectives, rather than what it is given. This implies the need for ongoing enquiry into health related social objectives (the numerous objectives loosely grouped under the heading of 'equity').*

### Practice variations

**Figure 1:**
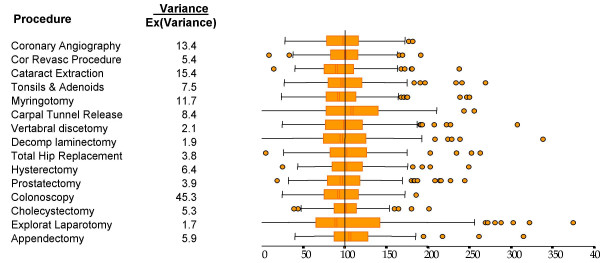
Standardised Rate Ratios for Various Operations in the Statistical Local Areas in Victoria, Compared to the Rate Ratios for All Victoria, Source: Richardson 1999 p198.

In 1982 John Deeble and I used data from the first full year of compulsory health insurance, (Medibank), to determine the level of service use in each of Australia's statistical divisions [23]. An example of the results is shown in Table 2. Huge variations in service use were detected even between the relatively large geographical units used in the study. Within these units small area variation would have further increased the discrepancies. These differences do not appear to have diminished with time. Thus, for example, Robertson and Richardson [24] found startling variation in the use of well-defined hospital procedures even after standardisation for age, sex and population. In this study data were collected for a two year period for each of Victoria's statistical sub-divisions. Procedural rates were expressed as a percentage of the rates which would be expected from the State average service use per age-sex cohort and from the demographic characteristic of the Statistical sub-division (SSD). Results are summarised in Figure 1. The bar, lines and circles give an indication of the frequency distribution of the procedure utilisation rates (ie the 25 and 75th percentiles (bars), two standard deviations (lines) and outlying SSDs).

**Table 2:** Practice Variations 1976

**Consultations per capita**			
**Statistical Division**	**GP/(GP)**	**Q(Spec)**	**Total**

Sydney	5.1	2.3	7.4
Tasmania	3.1	1.3	4.4
Darwin	1.1	0.5	1.6

Sydney/Darwin	4.6	4.6	4.6

The results reveal an 8 to 10 fold variation in service use. Part of this is attributable to the random variation that would be expected because of the uncertainty of the episodes of ill health. Using state-wide data the ratio of actual to expected variance was calculated. This is reported in the column of numbers to the left of the bar diagram. For the first procedure, coronary angiography, the observed variance was 13.4 times greater than expected; ie actual variance was 1,440 percent of the expectation. This extraordinary result is reproduced for all of the procedures examined.

A second example of uneven service delivery was also published by the same authors. This related to the likelihood of obtaining a high tech procedure – angiography or revascularisation – after a heart attack. Results shown in Table 3 indicate that in the 14 days following the heart attack, men and women admitted to a private hospital were 2.2 and 2.27 times more likely to receive angiography than their counterparts at a public hospital. They were 3.43 and 3.86 times more likely, respectively, to undergo revascularisation (coronary artery bypass surgery angioplasty, stent). These discrepancies did not diminish significantly in the following 12 months. The same study identified statistically significant differences in the likelihood of a procedure between men and women, young and old, urban and rural populations.

**Table 3:** Ratio: (Likelihood of procedure after admission to a private hospital) ÷ (Likelihood of procedure as a public patient)

	**Angiography**	**Revascularisation**
Within 14 days		
Men	2.20	3.43
Women	2.27	3.86
Within 12 months		
Men	2.16	2.89
Women	2.22	2.84

Taken together these studies suggest that there is a highly erratic pattern of service delivery across Australia and between social groups. One of three conclusions is inevitable. Some groups are under-serviced; some groups are over-serviced or both of these problems occur to different sub-groups of the population. This indicates both allocative inefficiency (more health could be obtained with a redistribution of existing resources) and significant inequity for those with poor access to health services or a different form of inequity for those persuaded to undergo procedures where risks exceed likely medical benefits. Government has shown almost no interest in this type of result. There has been recognition of an 'urban-rural' discrepancy but, 20 years after the demonstration of a more complex pattern than a simple urban-rural dichotomy, remarkably little has been achieved.

***Conclusion 14:**Significant variation in the use of services has been allowed to continue more than two decades after it was identified. Small area variation across Australia almost certainly reflects significant allocative inefficiency and an inequitable access to health services. There appears to have been relatively little interest in this problem.*

### Lack of coordination

The health system at present consists of a series of financially independent programs ('silos'), which are poorly linked to other health services or programs. There appears to be near universal agreement that a sensible health scheme cannot be built upon the current Federal-State division of financing, responsibilities and powers or upon the existing Commonwealth program structure. In the context of a recent review of hospital care, the Tasmanian Government accepted a recommendation for the pooling of all health and aged care resources [25]. However implementation of the recommendation requires Commonwealth support which may or may not be forthcoming. The fact that a simple solution exists for this most elementary problem but, to date, has not been seriously addressed, represents a fundamental failure of government in Australia.

Two case studies are used below to illustrate what gold standard allocative efficiency would imply for the use and coordination of services. They indicate the distance that health services reform must travel in Australia before we have gold standard delivery.

The first case study is real. A Seattle-based 'pure' Managed Care company, Ethix, was asked to establish a health scheme for a small town close to Seattle. Routine surveillance of the medical claims over the first two years of the new scheme highlighted an anomaly. There were excessively large numbers of youths receiving surgery for spinal injuries. Further investigation found that the problem was attributable to a toboggan run on the outskirts of the town which had a tree stump half way down the slope. Youths were crashing into the stump and damaging their spine. The health scheme paid for a bulldozer to remove the stump [26].

Medicare does not pay for bulldozer services. However, in the circumstances described here it should do so. More generally, the vignette illustrates two of the characteristics of gold standard delivery, namely, routine data surveillance to locate problems with any aspect of social or medical behaviour which might be modified to improve health and, secondly, the flexibility of funding which is needed to adopt the most cost effective solution to the identified problems. In contrast, in the Australian health scheme a problem of this sort would not be detected by the health system. It is possible that a similar type of accident could fall under the jurisdiction of an occupational health and safety or traffic accident authority. Otherwise there would be neither the will nor the means to respond. If such a problem was eventually identified the typical response would be accusation, blame shifting and, possibly, litigation. The cause of this problem is related to the practice of Management by Objectives, an aspect of managerialism, which encourages local rather than system-wide thinking. Interestingly, Peter Drucker, one of the early advocates of MBO, in recent years has publishes warnings about its over-use and limitations.

***Conclusion 15:**A key challenge is to establish a single payer (for each person) with flexibility and incentives to purchase the most cost effective services. Services should not be determined by historical program boundaries and rigid budgets. There has been a serious failure by government to address this fundamental issue. The achievement of this relatively simple reform is necessary (but not sufficient) for the achievement of a range of system reforms.*

The second case study is a hypothetical scenario constructed by Duckett to illustrate gold standard system coordination and processes [27].

'A woman with dizziness is concerned about her health. She rings the *State call centre *which advises her to visit her *local health team*. She is able to see the GP quickly who asks her a series of questions from the *relevant research based protocol *and undertakes a clinical examination. The GP emails the results to a local specialist... who orders some further investigations consistent with the *state research based care path*... *Advice *of (an) impending admission is automatically *conveyed electronically to the GP and the social worker in the referring health team*. The social worker contacts the hospital to *discuss discharge planning*... The specialist... suggest(s) a number of *sources for information about the patient's condition*. The *patient contacts the call centre for further information*... The case is *randomly selected by the hospital audit committee *for quality review. The committee suggests some slight *changes to the state-wide protocol committee*.' p204.

The key elements of this scenario relate to information access and transferral. It illustrates the role of evidence based medicine, routine service review, the adaptation of protocols, universal electronic transfer of all information and the absence of incentives to depart from best practice.

Parts of this scenario correspond with Australian practice. But the events in italics would be unusual. It appears to be serendipitous whether a particular problem of a particular person and in a particular part of Australia results in a response which even partially mirrors the gold standard response in this scenario.

***Conclusion 16:**Commonwealth and State authorities should mandate practices which improve the coordination of services. To facilitate this there should be universal use of electronic data systems for patient notes and information transfer. Evidence based protocols and clinical paths should be adopted when available and relevant. Feedback and error learning should be a routine part of the system. All of these desirable features are presently difficult because of the fragmentation of the system which is, in part, attributable to the failure to establish a single funder for all health services received by an individual.*

### Quality of care

Results from the 1995 'Quality in Australian Health Care Study' (QAHCS) suggest that the quality of health care in Australia is a problem which overshadows all others. In the initial study, reported by Wilson et al [28] medical records for more than 40,000 admissions to 28 hospitals in NSW and SA were individually examined to determine whether or not an adverse event (AE) was associated with the admission (prior to or during the episode of hospitalisation). A judgement was made concerning the consequences of the AE and whether or not it might have been avoided.

By extrapolating results the authors estimated that about 470,000 admissions were associated annually with an AE and that these would have resulted in 18,000 deaths and 50,000 cases of permanent disability. In a subsequent report Runciman et al [29] estimated that in 50 percent of the AEs in the QAHCS had a high preventability score. Sixty percent of deaths could have been avoided. In this latter study, incidence and not prevalence scores were reported as part of the effort to standardise the methodology with an earlier Harvard Medical Practice Study (HMPS) reported by Brennen et al [30]. This reduced the annual rate of AEs to 10.6 percent of admissions.

From the response to these events (discussed below) it appears likely that many have been unable to appreciate the scale of the carnage implied by the report. If the results from the original QAHCS are not discounted then medical errors have been responsible for the death of more Australians per annum than the average annual death rate of Australian soldiers in World War 1 (15,800). Permanent disabilities per annum approximate the annual rate of casualties in The Great War (62,500). Unlike The Great War, the problem of AEs has probably been ongoing for 50 years or more.

In Table 4 below the death rate from AEs is compared with mortality rates from other causes which are of particular social concern. To be conservative and to take account of undetected bias, the AE rate reported in a 1995 study is reduced by 50 percent. Preventable deaths are assumed to be 50, not 60, percent of deaths associated with an AE. The resulting, conservative number of deaths from AEs in 1999 were about 40 percent higher than the number of deaths from AIDS, suicide, motor vehicle accident, accidental falls, homicide, drowning and poisoning combined. In Table 5 some equivalent events are listed. The conservative estimate of the unnecessary death rate is about the same as would occur if the Bali bombing occurred every week of the year, year after year.

**Table 4:** Perspective: Selected Causes of death, 1999

**Cause**	**No of deaths**
AIDS	122
Suicide (intentional self harm)	2,492
Motor vehicle accidents	1,741
Accidental falls	520
Homicide	300
Accidental drowning/submersion	278
Poisoning by drugs/medications	1,015
Subtotal	6,468
All deaths from adverse events^(1)^	9,000
**All preventable adverse events^(2)^**	**4,500**

(1) 50 percent of reported estimate	
(2) assuming 50 not 60 percent are preventable	

**Table 5:** Events equivalent to avoidable AE deaths*

**Cause**
1 in 10 customers in restaurants poisoned each year: annual deaths 4,500
13 Jumbo jets crash each year, each with 350 Australian passengers killed
45 Bali bombing type attacks, each with 100 Australians killed each year
'September 11' every 8 months: only Australians die
*assuming preventable deaths = 25% QAHCS (1995)

While those affected by adverse events will, on average, be older and frailer than those who died in Bali or during World War 1, the magnitude of the problem is still staggering. Considering the reaction to the Bali bombing it might have been expected that the publication of the QAHCS would have caused a seismic shock, with the public demanding immediate, comprehensive reform and government passing urgent legislation to mandate any achievable system reform which ameliorated the problem. If the system failures which preceded the Bali bombing warrant a Parliamentary inquiry, equivalent interest might have been expected with respect to a problem responsible for an equivalent number of deaths every week. An effectively unlimited budget might have been approved for the upgrading of quality and safety.

In contrast, the actual reaction to the report must constitute one of the more puzzling episodes of Australia's social history. At all levels the response was sedate, cautious and incremental and there appears to have been greater concern that our health system might be perceived as unsafe than with the fact that it actually is unsafe. The level of activity in the 5 years following publication of QAHCS suggests that the results may not have been truly believed. But no subsequent study was funded to validate or refute the results.

The QAHCS results were not ignored. As summarised in Table 6 an advisory body was immediately established which evolved into the Australian Council for Safety and Quality in Health Care (ACSQHC). Its activities are summarised in five successive annual reports. In addition, the Australian Health Care agreement between the Commonwealth and State governments allocated budgets of $680 million and $785 million for quality assurance activities for the periods 1998–2003 and 2003–2008. In each of the States sub-committees and working groups were created which, along with the ACSQHC have resulted in a very large number of reports, publications, some legislation and local initiatives. The ACSQHC alone lists 35 publications [31]. State activities are summarised in ACSQHC, [32]. These initiatives have resulted, *inter alia*, in moves to tighten up hospital accreditation processes; to monitor adverse events more closely; to improve consumer participation in the evaluation of health care; to encourage health professionals to report adverse events; to improve health information technology; to establish practice guidelines, etc. The large number of funded projects are described and listed in ACSQHC, [33].

**Table 6:** Response to QAHCS

1995	QAHCS published
1996	Taskforce established on QAHC
1998	Health ministers ask Advisory Group for report
1998	Interim Report
1999	Report of the National Expert Group
	'Actions identified by the taskforce... need to be implemented at all levels of the health system ...'
1999	Australian Council for Safety and Quality in Health Care Established

Despite the high level of activity, the importance, priority and sense of urgency reflected appear more appropriate for an (important) ongoing reform process in an already well-operating system. There is a yawning gulf between this and what the public would undoubtedly demand if our TV and news services were reporting deaths on the scale of the September 11 New York disaster very two months accompanied by about three times this number of injuries. The philosophy of the reform process appears to be summarised by the ACSQHC when it approvingly quotes Berwick as saying that 'there are no quick fixes. We must re-examine all that we do', p14 [31].

As a description of history the 'no quick fixes' statement is correct. The 1999 report – 4 years after the publication of the QAHCS recommended that 'actions identified by the taskforce... need to be implemented at all levels of the health system'. By 2001 NSW had passed legislation which, *inter alia*, created the Institute of Clinical Excellence. By 2002 – 6 years after publication – the Victorian Quality Council Plan had been established. In 2003 the updated version of this plan set as its goals, *inter alia*, the establishment of a framework, the involvement of consumers and education. Almost 10 years after the report, the ACSQHC called for mandatory participation of all hospitals in a process of assessment [31] and (with possible irony or ill concealed frustration) the Chairman of the ACSQHC argues in the 2004 Annual Report that 'Action must be taken without untimely delay where culpability is clear', p5 [34].

Between publication of the QAHCS and the Health Ministers' request for a report in 1995, 13,500 Australian would have died and 37,500 become permanently disabled. By the time the 1999 report was recommending the implementation of various policies at least 18,000 would have died. By the time of the NSW legislation cumulative national deaths would have reached 27,000. The Victorian Strategic Plan to develop a framework was published after the death of at least 31,500.

It is scarcely surprising that in 1999 an editorial in the Medical Journal of Australia commented that:

'Welcome though (various initiatives) are, the pace of change nevertheless seems slow given the stark message of the original QAHCS study four years ago... 50,000 Australians suffer permanent disability and 18,000 die at least in part as a result of their health care', p404–50 [35].

By 2002 Siddons could still comment that:

'On the 10^th ^anniversary of the study year, the most striking outcome has been the paucity of reform currently exhibited at the coalface of tertiary health care', p823 [36].

The inadequacy of the response is surprising as the evidence suggests that a reduction in AEs would be spectacularly cost effective. The interim report from the national expert group estimated the potential savings from preventable adverse events in 1995/96 would be $4.17 billion *per annum*. Consequently, expenditures of this amount could be justified if they eliminated the unnecessary AEs or if the cost of the achievable 50 percent reduction in AEs was less than $2 billion *per annum*.

In contrast with the view that little could be done quickly there are a number of examples where, *prima facie*, very significant and effective change could have been/can be rapidly implemented. The chief distinguishing feature of each of the suggestions below is that they involve legislation and regulatory enforcement which appears to be inconsistent with the apparent emphasis upon persuasion and voluntary culture change in many of the present activities. Mandated options include the suggestions below.

***Accreditation***For decades health professionals have believed that a significant number of small hospitals are dangerous. However there has been no decisive action. With full knowledge of the QAHCS results, hospital accreditation remains voluntary in all States except Victoria. There will clearly be self-selection. Low quality hospitals will opt not to seek accreditation and poorly qualified doctors will seek out these hospitals. Universal accreditation could be mandated. Multiple accreditation teams could have the power to randomly inspect hospitals or units within hospitals and close those judged to be dangerous – as occurs with restaurants with sub-standard hygiene. It is unclear whether or not present accreditation is sufficiently rigorous to reduce avoidable adverse events significantly. There appears little reason why the accreditation process should not itself be reviewed to ensure that credentialed hospitals satisfy rigorous safety standards in their facilities and procedures.

***Doctor Accreditation***Patterns of private practice patterns are already subject to scrutiny in Australia. But the chief purpose is to detect medical fraud. Legislation could require the examination of practices to detect those which deviate significantly from evidence based guidelines constructed by the relevant Royal Colleges. When there is a known relationship between the small number of procedures carried out by a doctor and negative outcomes, as occurs with surgery, critical annual procedure rates may be established which trigger the provision of information to the doctor, the mandatory review of the practice and finally the dis-accreditation of the doctor for the conduct of these procedures. While it is true that some doctors take on the hard cases partial standardisation for case complexity is possible and this problem would obviously be taken into account by those conducting the review. The appropriate systems could be established in 1 to 2 years.

***Mandatory Disclosure and Error Learning***It is not compulsory for hospitals or doctors to register AE and routinely provide feedback to facilitate error learning. This means that the most important vehicle for improving quality and reducing patient risk is not compulsory. The opportunity for error leaning is almost certainly under-utilised in a large number of hospitals and probably ignored by the doctors whose performance most needs monitoring. Legislation might ensure the universality of this critical system reform. The adverse events register could and should be linked to doctors and appropriate threshold levels installed which sequentially trigger information feedback, review, and finally dis-accreditation of the doctor. It is likely that the first of these steps will be sufficient to effect satisfactory change. Despite this, 9 years after publication of the QAHCS the chairman of the ASCQHC notes that 'we have insufficient accurate data for fully appreciating the current size of the multiple causes of this problem ... we need the data from multiple sources, including incident monitoring systems, routine administration data sources and the use of screening tools to practically identify areas that may cause harm.' (p5) [34].

***Protection from litigation***The published research on 'high reliability organizations' suggests that it is wise to separate inquisitorial from punishment processes, such as dis-accreditation. Adverse events are unlikely to be reported if there is a financial incentive to hide the AE. For this reason legislative protection of doctors from the financial outcome of litigation is probably a prerequisite for a successful and comprehensive system of error learning. The consequences for a doctor associated with AE should be based upon medical criteria and uncoupled from the social mechanism for compensating patients. Legal protection alone is unlikely to improve the quality of the information used for error learning. Rather, it should be part of a package of requirements which includes penalties for the failure to report an AE.

***Information transfer***Patient notes are still transferred within hospitals using 19th Century clipboards. It is known that this commonly causes potentially lethal errors. The mandated use of (long available) electronic forms of transmission could alert staff to the risk of inappropriate procedures, the administration of conflicting drugs or the failure to administer a drug. Likewise X-ray films are often misplaced or lost. The consequences may be lethal. Legislation could ensure the use of digital technology to ensure immediate access of results. New wireless technologies make it possible for roving staff – doctors and other professionals – to have constant access to text and basic technical data. As with electronic note taking this might take 1 to 2 years to fully install in all Australian hospitals, clinics and nursing homes.

***Hospital systems***Hospital systems in Australia are commonly ramshackle or antique. There are no required pathways or mandatory discharge criteria. There are no internal or external financial incentives for the optimal treatment of patients. These changes are more complex but could be installed comfortably in 4–6 years.

***Conclusion 17:**There is no reason why much of the health system should have missed the IT revolution which has transformed other parts of the community. In relation to the size of the AE problem, the cost of implementing late 20^th ^Century information technology throughout the health system is likely to be small relative to the human and financial cost of AEs averted.*

***Minimum staffing requirements***There is no regulation which links on site expertise and the complexity or riskiness of the procedures which may be undertaken in a hospital. For example, it is possible for a hospital to permit significant surgery but have no on-site medical practitioner post-operatively. This potentially lethal practice could be proscribed and minimum staffing ratios implemented within the 1–2 weeks needed to reschedule staff or the location of procedures. It was not until 2003 that the ACSQHC released a paper considering issues of staff rostering, skill mix, staff numbers, staff supervision and team functioning [33]. While endorsing the AMA (voluntary) code of practice, [37] it comments – almost 8 years after the QAHCS – that 'responsibility for improving the management of staffing variables cannot (ie should not but still is being) left to individuals. It is a governance responsibility...' pii (words in brackets added)] [38].

***Queuing***The airline industry operates a highly efficient computerised system of booking and queuing which may be accessed by travel agents throughout the world. By this standard most hospital queuing systems – if they exist – are rudimentary. It is possible and desirable for different hospitals in the public hospital system to be interconnected to provide patients or their doctors with available times for treatment city, state or nation-wide. Queuing and scheduling can and should be operated using publicly known criteria. In the USA there is a nation-wide system for matching patients with available organs. (There is, interestingly a prioritisation criterion which, for reasons of equity, assigns compatible organs on the basis of need, not prognosis.) Australia has no such system.

***Conclusion 18:**Queuing should be regulated by a nation-wide prioritising system based upon explicit criteria. This would increase efficiency and, for the patient, increase choice and certainty.*

The private sector would have greater difficulty in operating such a system because of the patient's attachment to a particular doctor. Private health insurance organisations could, however, offer a similar service to patients who are willing to accept treatment from contracted providers. Australia's technologically conservative PHI organisations appear uninterested in these initiatives.

***Information and system audit***The suggestions above and, to date, the majority of the reforms contemplated in reports, represent process measures of success. However, their objective is to reduce adverse errors and for this reason, record analysis of the form conducted by the QAHCS should be an ongoing feature of the system. The QAHCS research was relatively expensive, but these costs are infinitesimal in relation to the importance of the surveillance, the costs, the morbidity and deaths averted.

***Conclusion 19:**A policy of persuasion and culture change is an insufficient response to a problem of the magnitude of the AE epidemic. As a matter of highest priority, legislation should be passed requiring extensive system regulation and reform to reduce the incidence of adverse events. The required changes should be fully funded (or they will not occur) and should include the use of state of the art IT, minimum staffing requirements and system feedback on both individual hospital and individual doctor performance. Evaluation and random audit of all hospitals and health care providers and the reporting of all AEs should be mandatory. Doctors should be given comprehensive protection against the results of successful litigation. The review of doctor performance should be uncoupled from compensation for the patient.*

***Public information***There is no legitimate reason for information relating to hospitals and individual doctors to be withheld from the public. There is a persuasive argument that the public has a right to such information. Additionally public awareness and the likely response from the public to such information are likely to accelerate the pace of reform. There are persuasive reasons for providing information more generally concerning the performance of all parts of the system, including individual care providers. Patient choice in the absence of information is a charade. Failure to forewarn patients that the hospital of their (doctors') choice has a substandard safety record and that capacity exists in hospitals with a better record could, and arguably should, be regarded as professional negligence. There can be little doubt that, if consulted, the public would overwhelmingly endorse the need for this information. More generally, the provision of information is an effective method for effecting change and it is unlikely that the pace of reform would have been as sedate if the public were properly aware of the safety record of various health care providers.

One argument against this option is that the provision of information might result in a loss of confidence in hospitals and doctors. However, the argument that the public should be kept in ignorance to engender unjustified confidence is, at best, dubious and if this ignorance allows an inadequate policy response then it is additionally harmful. In some states of the USA, and most notably New York, severity adjusted mortality rates are available for every hospital and for every doctor. This has not resulted in a significant change in the pattern for public demand but it has galvanised doctors and hospital staff to successfully review and upgrade their procedures [12]. League tables have recently been introduced in England to allow doctors and patients to evaluate the performance of particular hospitals [39]. From late 2004 the performance of individual surgeons will probably be available [40].

***Conclusion 20:**Information is a key element in achieving system reform and for the efficient operation of all markets, private or public. All institutions and individuals should receive rapid feedback on their performance. Information should be routinely collected and also obtained from random audit of hospitals. In particular, information should be publicly available with respect to the safety record of hospitals and providers of medical care.*

***Financial incentives***As discussed earlier, financial incentives are one some of the most effective, non-coercive ways of achieving desired outcomes. There has been very limited use of this powerful instrument and the financing of medical services has generally been perceived as a reward for providers doing what they select to do rather than as an opportunity for influencing what is done. This is an important missed opportunity. Importantly, financial incentives are non-coercive and avoid the head-to-head conflict between 'clinical autonomy' and the 'patient's right to evidence based medicine' which may accompany direct regulation.

***Conclusion 21:**Financial incentives should be used flexibly throughout the health system to induce behaviour which minimise AEs.*

***Governance and ownership of the problem***A key theme of this paper has been that government policy has focused almost exclusively upon financial issues and that there has been little concern from within the legislature with health services and health. This is possibly the root cause of the government failure. Activities from within the various bureaucracies have largely been bureaucratic. In its 2004 review of State initiatives the ACSQHC reports that Queensland now has 'a program to develop a workforce culture that values a multi-disciplinary evidence based approach to improvement'; in 2003 the NT implemented an 'overarching quality committee'; NSW had 'involved the appointment of patient safety managers'. The ACT now has a framework which 'provides explicit lines of accountably'; Victoria now 'has a policy of an open and transparent approach to the provision of information' etc (p8) [34]. There is no reference to national legislative action to enforce safety.

The ACSQHC itself appears frustrated with the scale of the national effort when it comments that 'over the last year with public failures reported in some Australian hospitals it is clear that a small program of national investment and development needs to be matched by the will, skill and capacity of stakeholders....' (p5). On the same page it comments that 'Council is actively working to further develop data sources, but cannot do so without continued support by all governments' (p5). Almost 10 years after the 1995 report the ACSQHC comments that 'future work ... needs to consider what governance and responsibilities are required at all levels of the health care system to ensure the provision of safe health care' (p84).

***Conclusion 22:**Despite the unnecessary death of between 40,000 and 80,000 Australians since the publication of the QAHCS, Australian government has not taken ownership of the problem of adverse events. As a result, those attempting to effect change have been forced to 'coax and cajole' Options to 'mandate and enforce' have been largely ignored.*

### Disregard of evidence

The last conclusion relates to the need to generate and disseminate information relating to system safety. The arguments for this apply more generally to health system data. Australia has a wealth of administrative databases which could be employed to investigate and improve system performance. As noted earlier, service use is very uneven across Australia. To the extent that this violates the usual notion of 'equity' this information should be an important input into policy formulation. It does not appear to be used for this purpose outside NSW. However the quality of the data also presents other opportunities. The effect of different treatments, such as the varying rates of angiography and revascularisation observed between public and private hospitals could be traced through time if record linkage were possible. This would allow an assessment of downstream costs, mortality and morbidity associated with the two patient groups. There are clearly many opportunities for longitudinal research of this sort. A further option which may be piggy-backed on administrative data is the routine provision of information to different groups of patients who have been identified by their service mix. Information of this sort is probably a highly effective way of 'empowering patients'; that is, enabling patients, and particularly those with a chronic disease, to take greater control of their disease management.

These developments have not occurred for a variety of reasons. First, for an $80,000 million industry, research funding for 'product development and marketing' – health services research – is astonishingly small. In the USA six Federal agencies alone spent $US 1,658 million in 2002 upon HSR. The agencies and their expenditures are detailed in Table 7. Significant US funding is also obtained form the US network of Foundations which does not exist in Australia. Benchmarking against the Federal agencies alone, at an exchange rate of $US 0.70 = $AUS 1.0 (0.65) and scaling these expenditures down in relation to the size of the US and Australian economies, Australia should be spending about $AUS 120 million on HSR. Australia does not currently spend a fraction of this amount. As a major initiative, the NHMRC is to provide $10 million per annum for HSR – or about 7.7 percent of the US Federal benchmark.

**Table 7:** US expenditure upon Health Services Research

**Federal Agency**	**Expenditure ($US millions)**
Agency for Health Care Research and Quality	300
National Centers for Health Statistics	127
Extra Mural Prevention Research CDC	18
Centers for Medicare and Medicaid Services	55
Veteran's Health Administration	371
National Institute of Health	787

Source: Annual reports

A second and possibly related reason is that there is no dedicated instrumentality, similar to the AIHW, which has taken 'ownership' of the need to provide and periodically to review the need for information generated by HSR. Funding is currently inadequate but also ad hoc.

Third, concern over the confidentiality of records has been elevated to such a level that easy and routine data linkage to observe the outcome of different service patterns does not seem to be a possibility. For example, access to Australia-wide, de-identified public hospital records requires the separate consent of all States and Territories as well as the cooperation of the Commonwealth Department of Health or AIHW. Data linkage to determine the consequences of different treatment patterns – who lives and who dies – is so difficult that the research is effectively proscribed.

It is extremely doubtful that this concern in the bureaucracy over privacy would reflect the preferences of a well-informed population. Patients almost certainly suffer and die because of the interpretation and implementation of our confidentiality laws in a way which seriously inhibits the capacity of the public, researchers and private and public agencies to investigate the outcome of system performance and differences in individual treatments. The bureaucratic fetish with confidentiality does not occur in the USA where the risk of litigation is significantly greater than in Australia. In some states data relating to hospital and doctor mortality rates are regularly published and in California unit record hospital data is available on CD in university libraries.

***Conclusion 23:**Routinely collected administrative data should be fully used to monitor system performance. In particular it should be employed to monitor equity of access to services regularly and to provide disease-related information to population groups identified as having particular needs and interests. A statutorily independent national institute for health services research should be established whose terms of reference require the achievement of these objectives.*

## 5 Discussion and conclusion

There are various options for the macro reform of the health system and the corresponding reform of financial incentives and the roles and responsibilities of the various players. In particular, Dick Scotton has cogently argued for the adoption of Managed Competition [41-43]. A partial movement in this direction could be achieved by transferring responsibility for the purchase of health services to the various health regions [44]. There has been no attempt to review this large topic here. Rather, the paper has reviewed the 'micro' elements of such reform. The most appropriate 'macro' model for the health system is the model which maximises the likelihood of implementing satisfactory solutions to the numerous problems facing the system including those that have been discussed above.

The chief conclusion from this paper is that the 'health care debate' and recent policy 'reform' has focussed upon issues which are best described as 'very small order' as judged by their likely effect upon either health or the cost effectiveness of the health system. They have been primarily concerned with dollars, not health and, more specifically, with the distribution of the cost between the public and private sectors.

None of the policies discussed in Section 3 of the paper is likely to increase cost effectiveness. Cost shifting from the PBS to the public creates differential copayments which encourages allocative inefficiency. PHI reforms have created an industry with bizarre financial incentives and is a spectacular example of negative micro economic reform.

The common feature of the three policies is that each moves the health system in a direction which is more consistent with the liberal/libertarian world view in which responsibility is transferred to the individual and away from the community. While PHI helps the individual to avoid financial decisions at the point of service, the individual is responsible for the purchase of the insurance and for the payment of (net) premiums. Copayments are the most direct method for shifting responsibility to the individual users of health services. Bulk billing for pensioners/health care card holders preserves the safety net which is needed for the achievement of equity as commonly defined by this world view. Over the longer term the pursuit of these values would redistribute income to the healthy, wealthy and away from the unhealthy unwealthy which is the antithesis of the communitarian/solidarity value system. The vehicle for the transfer is both a decline in community financed expenditures and a corresponding decline in taxation.

As discussed in Section 2, decision making with respect to social objectives is the legitimate role of government. However, good economic and social policy seeks to achieve these objectives in a way that is cost effective. The reforms discussed here have not achieved this.

In contrast with these policies, the five neglected areas discussed in Section 4 deal with problems which are 'large order issues' as judged by their likely impact upon health and the cost effectiveness of the health system. Also contrasting with the first group, these issues are relatively 'value neutral' as judged by either the communitarian or libertarian perspectives. The failure to address satisfactorily these issues is attributable to neglect, not the dominance of a particular ideology. This failure over a very long period itself refects a failure in the governance structure and a failure to identify and act upon opportunities for quantitatively large system improvements. The failure is probably replicated in most other developed countries, reflecting the complexity of the health sector. But benchmarking against similarly impaired systems does not alter the fact that there are opportunities for significantly improving the community's health which have been largely ignored. The reasons why this has occurred has not been discussed here and, to a greater or lesser extent, these failures have probably been replicated in most other developed countries, reflecting similar social and technical histories.

## Competing interests

The author(s) declare that they have no competing interests.
